# OR6-001 - S100A12 as pro-inflammatory TLR4 ligand

**DOI:** 10.1186/1546-0096-11-S1-A96

**Published:** 2013-11-08

**Authors:** C Kessel, H Wittkowski, A Lüken, T Weinhage, G Varga, T Vogl, T Wirth, D Viemann, P Björk, M van Zoelen, F Gohar, G Srikrishna, J Roth, D Foell

**Affiliations:** 1 Pediatric Rheumatology, Clinical-Translational Innate Immunity Research, WWU Medical Center, Münster, Germany; 2 Institute of Immunology, WWU Medical Center, Münster, Germany; 3 Department of Pediatric Pulmonology, Allergology and Neonatology, Hannover Medical School, Hannover, Germany; 4 Active Biotech, Lund, Sweden; 5 Department of Experimental Medicine, Academic Medical Center, University of Amsterdam, Amsterdam, the Netherlands; 6 Sanford-Burnham Medical Research Institute, La Jolla, CA, USA

## Introduction

The granulocyte-specific Ca^2+^-binding protein S100A12 is overexpressed during autoinflammation as well as other inflammatory conditions in humans and has been ascribed to the group of pro-inflammatory Damage Associated Molecular Pattern molecules (DAMPs). S100A12-levels in human serum reveal excellent correlation with patients’ inflammation state, which renders S100A12 a powerful biomarker. In order to operate as DAMP S100A12 requires binding to cellular receptors. The protein was originally found to bind RAGE and was in turn entitled extracellular newly identified RAGE-binding protein (EN-RAGE). RAGE ligation by S100A12 is proposed to trigger a pro-inflammatory cascade in microvascular endothelial cells, macrophages and lymphocytes, culminating in NFκ-B activation. This amplifies the inflammatory response by triggering further RAGE expression and thus drives a feed-forward loop that can potentiate inflammation.

## Objectives

RAGE-binding by S100A12 can still be discussed controversial and an alternate receptor for S100A12 on mononuclear cells is suggested. Based on the previous discovery of S100A8/A9 signalling through TLR4 we studied the relevance of this particular pathway for S100A12 as alternative to the originally postulated signaling through RAGE.

## Methods

The release of human S100A12 from granulocytes as well as the promotion of inflammation by activation of human monocytes after specific receptor-interaction was investigated by a series of *in vitro* experiments on primary cells and receptor-expressing cell lines.

## Results

Upon inflammatory challenge S100A12 expression from human granulocytes is rapidly induced *in vitro* and *in vivo*. The protein is both passively released from necrotic cells and secreted via alternative secretory pathways. A global gene expression analysis of S100A12-activated monocytes revealed that human S100A12 induces strong inflammatory responses. These can be abrogated by specifically blocking toll-like receptor 4 (TLR4) on primary human monocytes as well as TLR4-overexpressing HEK-TCM cells. On the contrary, blocking S100A12 binding to RAGE reveals no such pronounced effect on both monocytes and RAGE-overexpressing cell lines. Importantly, as the observed effects on human monocytes appear to be TLR4-dependent, the S100A12-induced gene expressing pattern differed in part significantly from that induced by the primary TLR4 ligand LPS.

## Conclusion

We identified human S100A12 as an endogenous TLR4 ligand that induces a unique pro-inflammatory gene expression signature resulting in monocyte activation. Beyond the well-documented implication of S100A12 as inflammatory biomarker, our data shed new light on the role of S100A12 as powerful amplifier of innate immunity during inflammation.

## Disclosure of interest

None declared

